# Effects of visual-electrotactile stimulation feedback on brain functional connectivity during motor imagery practice

**DOI:** 10.1038/s41598-023-44621-6

**Published:** 2023-10-18

**Authors:** Chatrin Phunruangsakao, David Achanccaray, Saugat Bhattacharyya, Shin-Ichi Izumi, Mitsuhiro Hayashibe

**Affiliations:** 1 Neuro-Robotics Laboratory, Graduate School of Biomedical Engineering, Tohoku University, Sendai, Japan; 2 Presence Media Research Group, Hiroshi Ishiguro Laboratory, Advanced Telecommunications Research Institute International, Kyoto, Japan; 3 School of Computing, Engineering and Intelligent Systems, Ulster University, Northland Road, Londonderry, BT48 7JL UK; 4 Department of Physical Medicine and Rehabilitation, Graduate School of Biomedical Engineering, Tohoku University, Sendai, Japan; 5 Department of Robotics, Graduate School of Engineering, Tohoku University, Sendai, Japan

**Keywords:** Brain-machine interface, Biomedical engineering, Computational neuroscience

## Abstract

The use of neurofeedback is an important aspect of effective motor rehabilitation as it offers real-time sensory information to promote neuroplasticity. However, there is still limited knowledge about how the brain’s functional networks reorganize in response to such feedback. To address this gap, this study investigates the reorganization of the brain network during motor imagery tasks when subject to visual stimulation or visual-electrotactile stimulation feedback. This study can provide healthcare professionals with a deeper understanding of the changes in the brain network and help develop successful treatment approaches for brain–computer interface-based motor rehabilitation applications. We examine individual edges, nodes, and the entire network, and use the minimum spanning tree algorithm to construct a brain network representation using a functional connectivity matrix. Furthermore, graph analysis is used to detect significant features in the brain network that might arise in response to the feedback. Additionally, we investigate the power distribution of brain activation patterns using power spectral analysis and evaluate the motor imagery performance based on the classification accuracy. The results showed that the visual and visual-electrotactile stimulation feedback induced subject-specific changes in brain activation patterns and network reorganization in the $\alpha$ band. Thus, the visual-electrotactile stimulation feedback significantly improved the integration of information flow between brain regions associated with motor-related commands and higher-level cognitive functions, while reducing cognitive workload in the sensory areas of the brain and promoting positive emotions. Despite these promising results, neither neurofeedback modality resulted in a significant improvement in classification accuracy, compared with the absence of feedback. These findings indicate that multimodal neurofeedback can modulate imagery-mediated rehabilitation by enhancing motor-cognitive communication and reducing cognitive effort. In future interventions, incorporating this technique to ease cognitive demands for participants could be crucial for maintaining their motivation to engage in rehabilitation.

## Introduction

Motor paralysis is a serious issue that can have significant impacts on an individual’s quality of life, daily activities, and economic status^1^. Among several motor rehabilitation approaches, motor imagery-based brain–computer interface (MI-BCI) has gained widespread attention in recent years^2^. MI is a cognitive process in which an individual rehearses a motor task mentally without physical execution. Previous research has shown that the neural activity patterns observed during MI tasks are similar to those observed during actual physical movements, and that practicing MI can lead to cortical changes similar to those induced by physical practice^3,4^. Therefore, training the brain through MI can help patients regain motor functions by enhancing an individual’s ability to plan, execute, and control movements^5^.

Neurofeedback can be integrated into MI-BCI training by providing real-time sensory signals that reflect the neural activity of individuals^6^. This approach can promote neuroplasticity, which is the brain’s ability to adapt and reorganize in response to a new environment, in specific neural patterns^3^. This can result in improved classification accuracy^7^, increased perception of embodiment^8,9^, reduced cognitive workload^10,11^, and enhanced learning^12,13^. Additionally, modulating brain activity in specific patterns can help individuals to better control their own brain activity, leading to more effective and efficient use of BCI systems. While there is a general consensus that neurofeedback is essential for successful MI-BCI, there is still limited understanding of how neurofeedback modifies neural activity and brain connectivity^14^.

There are several methods used to observe neuroplasticity in the brain, including spectral analysis and event-related desynchronization (ERD) analysis^15,16^. Spectral analysis is a mathematical technique that utilizes the Fourier transform to analyze the frequency content of signals, with the most common approach being the analysis of the variation of power spectral density (PSD) or relative power (RP) of a frequency band of interest. ERD, on the other hand, involves the decrease in neural oscillation power in response to a time-locked event, such as MI, and has been shown to promote motor recovery and rehabilitation by facilitating changes in brain connections and the development of new neural pathways^3,17,18^. However, it is important to note that these methods do not provide a complete understanding of the complex reorganization and communication patterns within the brain network.

Healthcare providers can enhance the effectiveness of rehabilitation interventions and improve patient outcomes by understanding the neural mechanisms, specifically reorganization of brain network topology, that contribute to recovery. In recent years, there has been a growing body of evidence suggesting that the brain functions as a networked system comprising multiple specialized and spatially distributed areas that dynamically integrate information^19–24^. As a result, graph theory is increasingly being used to study and characterize complex brain networks. It offers a powerful tool for studying the complex connectivity patterns in the brain and identifying the critical regions necessary for information processing and communication.

Functional connectivity (FC) is a widely-used approach to modeling brain networks, where temporal correlations or statistical dependencies between different regions of the brain are represented using graph theory. This enables researchers to perform statistical comparisons of brain networks, both within and between groups of participants, before and after receiving treatment or stimulation^21,25–27^. To compare brain networks, researchers can employ various types of analyses. Edgewise analysis focuses on connections between different regions, such as their strength or efficiency, or changes in connectivity patterns. Nodewise analysis involves examining individual regions within the network, such as their degree or betweenness centrality. Network-wise analysis looks at the overall organization and properties of the entire network, such as its modularity or global efficiency.

The thresholding problem refers to the challenge of determining the appropriate threshold to remove weak connections between brain regions when constructing a graph representation of the brain^28^. The lack of a standardized approach to determine the suitable threshold can lead to inconsistent findings and either oversimplified or overly complex networks if the threshold value is too high or too low, respectively. To address this issue, a promising approach is to utilize a minimum spanning tree (MST) to represent brain networks. MST is a unique acyclic subgraph that connects all nodes and maximizes a specific property, such as synchronization strength between brain areas, resulting in a less biased network construction^29,30^. In recent years, researchers have employed the MST technique to study neurological disorders^22,31,32^, brain’s neuroplasticity^33^, and MI^34,35^, due to its sensitivity to subtle differences in brain networks compared to traditional methods.

Different modalities such as visual, haptic, and audio feedback can be utilized in neurofeedback training^36^. Employing a variety of these modalities in MI training can offer a more complete strategy to enhance brain training and motor function. Each modality has its benefits and can address diverse brain functions. Additionally, using a combination of modalities can increase the efficacy of MI training by presenting several feedback channels that can reinforce and complement each other^8,10,11,37^. However, in many studies, researchers have only used spectral, ERD, and classification analyses to examine how feedback can induce neuroplasticity. These methods do not provide a direct understanding of the complex interactions that occur between different regions of the brain. Therefore, this study aims to systematically investigate the changes in the topology of the brain network during MI practice in the $\alpha$ band by utilizing a graph-based FC approach in conjunction with conventional methods. This study investigates the effectiveness of different stimulation modalities by comparing the organization of the brain network during pre-feedback (no feedback provided) and feedback (feedback provided) sessions while participants performed left and right MI tasks. The study includes two groups of participants, one receiving visual stimulation (VIS) feedback and the other receiving a combination of visual and electrotactile stimulation (VES) feedback.

## Results

### Power spectral

**Figure 1:**
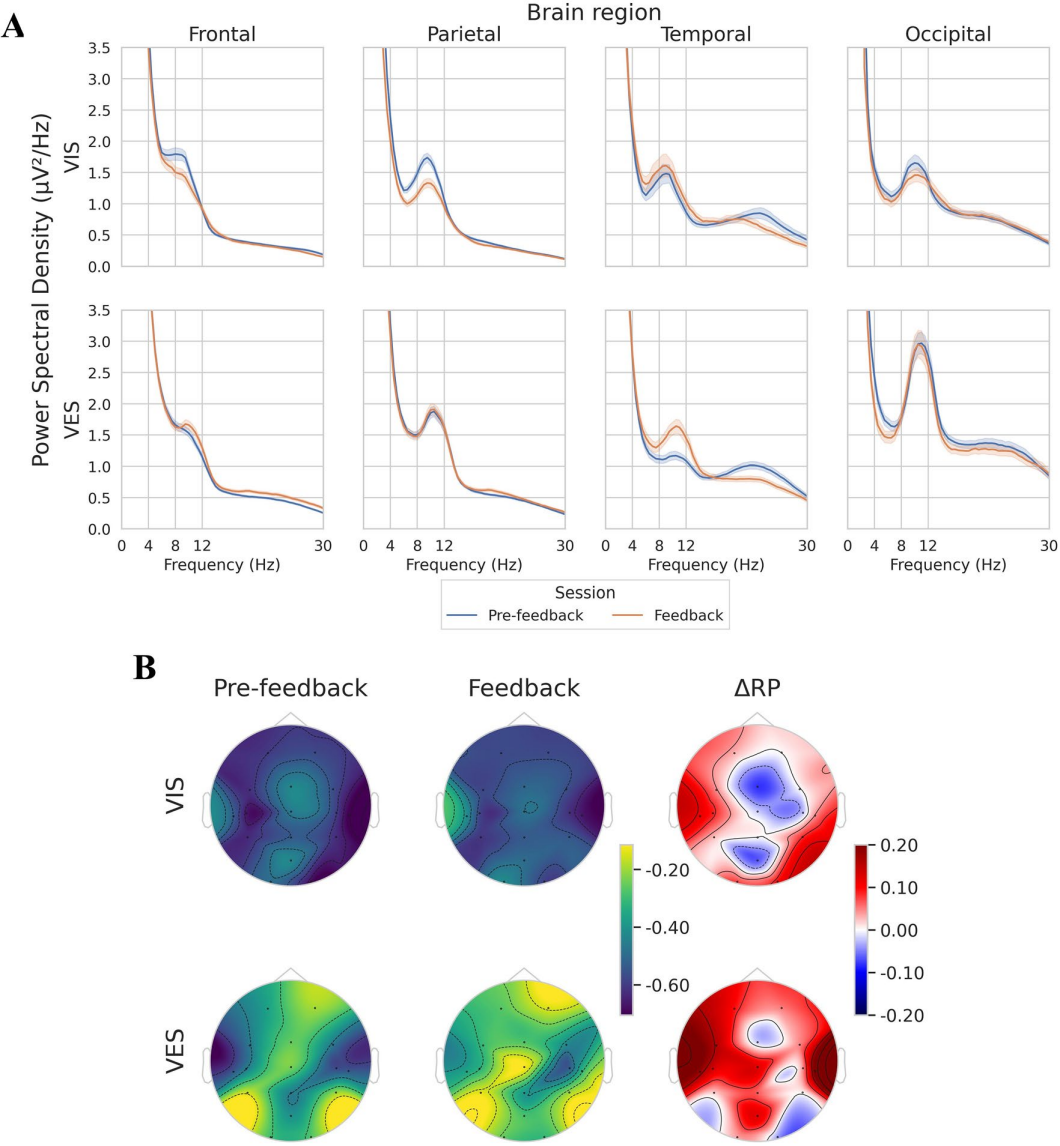
(**A**) Power spectral density (PSD) plot of four different brain regions (Frontal: AF3, AF4, FC3, FCz, FC4, Cz; Parietal: C3, C4, CP3, CPz, CP4, Pz; Temporal: T7, T8; Occipital: O1, O2) for each group. Both groups exhibited comparable power distributions across all frequencies. However, during the feedback session, the VIS group displayed slightly lower $\alpha$ band activity in the frontal and parietal lobes compared to the pre-feedback session. On the other hand, the VES group exhibited notably higher $\alpha$ band activity in the occipital lobe than the VIS group during both the pre-feedback and feedback sessions. (**B**) Topographic map of $\alpha$ band relative power (RP) for each group, where $\Delta$RP indicates the difference in RP for each comparison. There were statistically significant differences in $\alpha$RP in the VIS group, specifically in AF3, CP3, CPz, and Pz electrodes. In contrast, the VES group showed statistically significant differences in $\alpha$RP in C3, Cz, C4, Pz, and O1 electrodes.

The power spectral density (PSD) plot in Fig. 1 shows the frequency components of the EEG signals recorded during motor imagery tasks from four regions of the brain: frontal (AF3, AF4, FC3, FCz, FC4, Cz), parietal (C3, C4, CP3, CPz, CP4, Pz), temporal (T7, T8), and occipital (O1, O2). Although the VIS and VES groups exhibited comparable power distributions across all frequencies, the VIS group showed slightly lower $\alpha$ band activity (8–12 Hz) in the frontal and parietal lobes during the feedback session compared to that in the pre-feedback session. Additionally, both groups showed an increase in the $\alpha$-band activity in the temporal regions and a decrease in the $\beta$-band activity (12–30 Hz). The most obvious difference between the two groups was observed in the occipital lobe, where the VES group exhibited higher $\alpha$ band activity in both sessions. Because most of the observed differences occurred in the $\alpha$ band in different areas of the brain, subsequent analysis will mainly focus on this frequency band.

Frontal alpha asymmetry (FAA) was computed to measure emotional engagement during the neurofeedback training. The results showed that a slightly left-sided FAA was observed in the pre-feedback session (VIS: − 0.0606 ± 0.1935; VES: − 0.0620 ± 0.1502) and feedback sessions (VIS: − 0.0545 ± 0.2255; VES: − 0.0585 ± 0.1572) in both groups. Although both groups exhibited reduced left-sided FAA during the feedback session, the difference was not statistically significant (VIS: *p* = 0.8813, VES: *p* = 0.6250).

Figure 1B shows the topographic map of $\alpha$RP for each group. The results showed that the VIS group had a statistically significant reduction in parietal areas, such as CP3 (*p* = 0.0221), CPz (*p* = 0.0002), Pz ($p\ll$ 0.05), and AF3 ($p\ll$ 0.05). In contrast, the VES group showed a statistically significant reduction in the motor cortex, including C3 (*p* = 0.0104), Cz (*p* = 0.0001), C4 ($p\ll$ 0.05), Pz (*p* = 0.0022), and O1 (*p* = 0.0471).

### Brain network analysis

#### Edgewise

Edgewise analysis examines the connections between different regions of the brain and provides valuable information regarding the patterns of communication and integration between them. By analyzing the strength and properties of individual edges, researchers can identify the connections that play crucial roles in specific brain processes and behaviors. This study used network-based statistics (NBS) and graph edit distance ($d_{GED}$) for the edgewise analysis. NBS searches for a specific pattern or set of edges that distinguish the brain functional connectivity between two conditions^27^. In contrast, $d_{GED}$ quantifies the dissimilarity or difference between two graphs.

**Figure 2:**
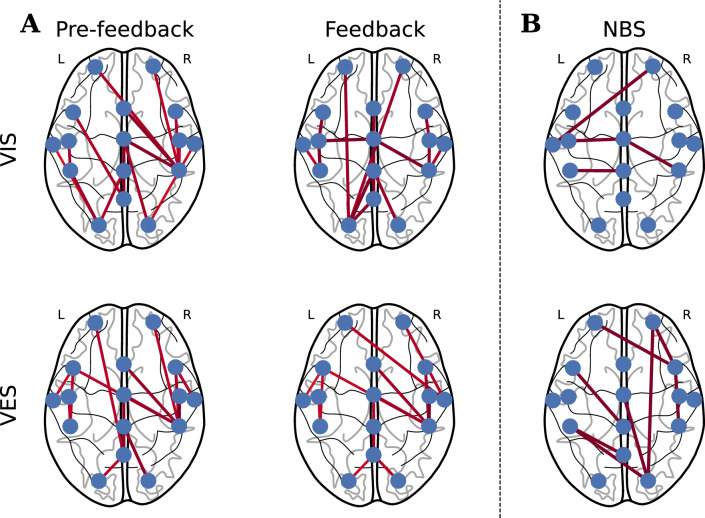
Plot of brain network organization based on functional connectivity in the $\alpha$ frequency band. (**A**) Group-specific connections computed by taking the average of functional connectivity across all participants prior to computing the minimum spanning tree (MST). (**B**) Significant edges between sessions, which were identified using network-based statistics (NBS). The left hemisphere is denoted by L and the right hemisphere is denoted by R. The VIS group had three subnetworks, mainly located in the parietal areas. Two significantly different subnetworks were identified in the VES group, with hub nodes located at FC4 and O2.

Figure 2A shows the group-specific brain network connectivity patterns under different experimental conditions. In particular, the VIS group exhibited more edges connected to the left occipital lobe and fewer edges connected to the right parietal lobe during the feedback session. In contrast, the VES group demonstrated relatively stable group-specific network connectivity across conditions. However, when averaging functional connectivity across all participants prior to constructing the MST for group-specific connectivity, there may be a loss of individual variability and important information. Therefore, it is recommended to avoid comparing group-specific connectivity directly and use NBS instead to assess edgewise differences more accurately.

The significant edges were determined using NBS with a t-value threshold of two, as shown in Fig. 2B. For the VIS group, three subnetworks were identified, mainly in the parietal areas, with some edges spanning the right hemisphere. Two sub-networks were identified in the VES group. The first subnetwork comprised eight nodes and seven edges with the hub nodes located at FC4 and O2. FC4 and O2 each had an edge connected to a node located in the opposite hemisphere, specifically AF3 and CP3. Additionally, both nodes shared a common edge connected to AF4. FC4 and O2 were connected to spatially close (AF4 and CP4) and spatially distant (AF4 and Cz) regions, respectively. The second subnetwork included an edge between FC3 and CPz.

**Figure 3:**
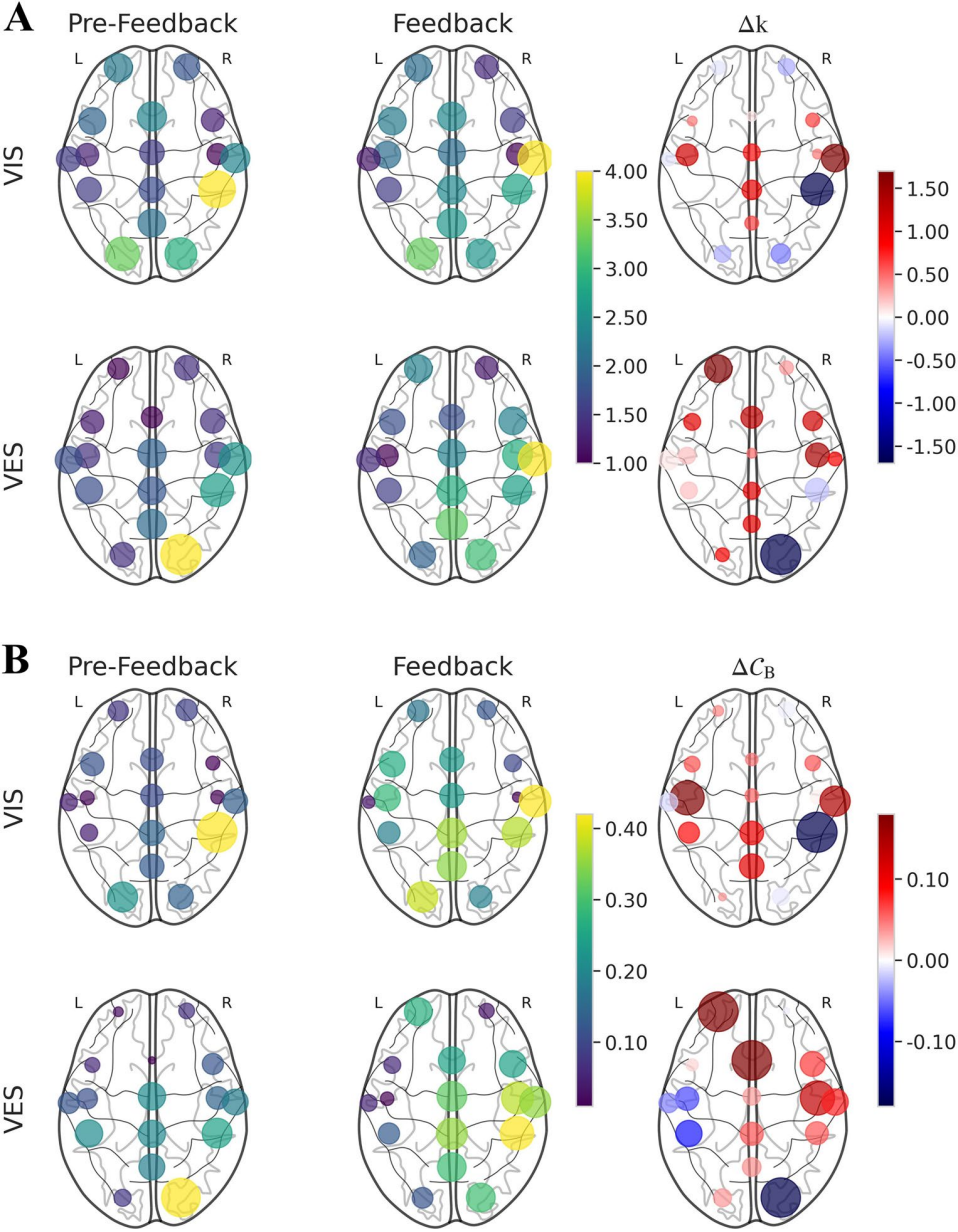
(**A**)Visualization of degree (*k*) for each group, where $\Delta k$ is the difference of *k* for each comparison, L denotes left hemisphere, and R denotes right hemisphere. The VIS group had subtler changes with increases at T8 and a decrease at CP4, whereas the VES group had large increases in *k* at AF3, but a decrease at O2. (**B**) Visualization of betweenness centrality (${\mathscr {C}}_{\textrm{B}}$) for each group, where $\Delta {\mathscr {C}}_{\textrm{B}}$ is the difference of ${\mathscr {C}}_{\textrm{B}}$ for each comparison, L denotes left hemisphere, and R denotes right hemisphere. The VIS group considerably more subtle changes with increases at C3 and T8 and a decrease at CP4, while the VES group showed significant increases in ${\mathscr {C}}_{\textrm{B}}$ at AF3, FCz, and C4 but a decrease ${\mathscr {C}}_{\textrm{B}}$ at O2.

**Figure 4:**
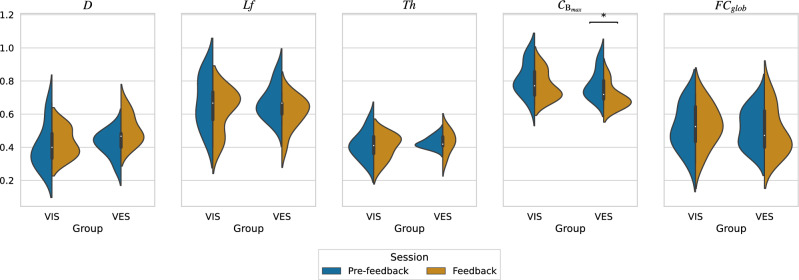
Violin plot of MST metrics (*D*: diameter, *Lf*: leaf fraction, *Th*: tree hierarchy, ${\mathscr {C}}_{\textrm{B}_{max}}$: maximum betweenness centrality, $FC_{glob}$: global functional connectivity) for each group. Asterisks placed above the corresponding conditions indicate statistical significance ($p<$ 0.05; FDR-corrected). Both groups showed an increase in *D* and a decline in *Lf* between sessions, indicating a decrease in the efficiency of information flow and network centralization, respectively. Both groups also had similar *Th* values, suggesting a star-like topology, and showed no significant difference in $FC_{glob}$. However, only the VES group exhibited a statistically significant decrease in ${\mathscr {C}}_{\textrm{B}_{max}}$, indicating a reduced reliance on a single node for information transfer.

We examined the distribution of $d_{GED}$ between the sessions. The VIS group (11.40 ± 4.22) had a lower mean $d_{GED}$ and narrower distribution of values compared to the VES group (13.80 ± 5.20). Additionally, we compared the subject-specific graphs to examine the consistency of the alterations, and the resulting distances were averaged to calculate the final $d_{GED}$. The VIS group had a higher $d_{GED}$ for both the pre-feedback (VIS, 17.07; VES, 14.89) and feedback sessions (VIS, 17.91; VES, 15.69). Furthermore, there was an increasing trend in $d_{GED}$ from the pre-feedback to the feedback session in both groups. However, the VES group demonstrated less intrasubject inconsistency than the VIS group in both sessions.

#### Nodewise

In the nodewise analysis, the degree (*k*) and betweenness centrality (${\mathscr {C}}_{\textrm{B}}$) were calculated for each node in a graph. Although both measures offer valuable insights into the structure and function of a network, and aid in identifying significant nodes or subgroups within the network, they capture distinct aspects of the importance of nodes. *k* represents the number of edges directly linked to a node, whereas ${\mathscr {C}}_{\textrm{B}}$ represents the significance of a node in connecting different parts of the network by identifying how often it appears on the shortest path between two nodes in the network. The *k* and ${\mathscr {C}}_{\textrm{B}}$ values for each electrode site are displayed in Fig. 3 using circles with different sizes and colors indicating the corresponding values.

The results from *k* and ${\mathscr {C}}_{\textrm{B}}$ analyses reveal a similar trend. The VIS group demonstrated subtle changes compared to the VES group, including a substantial increase in node importance at C3 and T8 and decrease at CP4. In contrast, the VES group showed a large increase in node importance throughout the brain regions, with the most notable increase observed in AF3, FCz, and C4. However, a significant decrease in node importance was observed for O2. None of the nodes demonstrated any significant differences for either metric based on the statistical analysis.

#### Network-wise

Network-wise analysis was conducted to obtain a holistic understanding of the structure and operation of the brain network. In this study, various parameters such as diameter (*D*), leaf fraction (*Lf*), tree hierarchy (*Th*), maximum betweenness centrality (${\mathscr {C}}_{\textrm{B}_{max}}$), and global functional connectivity ($FC_{glob}$) were used to describe how the organization of the brain network is altered through different types of neurofeedback, as depicted in Fig. 4.

In the feedback session, both groups showed an increase in *D* compared with the pre-feedback session. Similarly, both groups showed a decrease in *Lf* between sessions. The differences in the *Th* values between each condition were small, and the values were mostly approximately 0.4, indicating a star-like topology. However, statistical analysis revealed no significant differences in these metrics.

The node with the highest value of ${\mathscr {C}}_{\textrm{B}_{max}}$ is considered critical for maintaining efficient communication between the different parts of the network. In this study, the mean and standard deviation of ${\mathscr {C}}_{\textrm{B}_{max}}$ for both groups decreased similarly between sessions; however, the VIS group exhibited a higher mean and standard deviation for both sessions. The statistical analysis revealed that only the VES group exhibited a significant difference in ${\mathscr {C}}_{\textrm{B}_{max}}$ (*p* = 0.0138).

This study also investigated $FC_{glob}$, a measure of the overall strength of connectivity in the brain network. The values of $FC_{glob}$ were found to be quite similar across all conditions, with the VIS and VES groups displaying a slight increase and decrease in mean $FC_{glob}$, respectively. However, the statistical analysis did not reveal any significant differences.

### Decoding

Table 1 shows the ShallowConvNet^38^ decoding performance for each participant in terms of classification accuracy and Cohen’s kappa value, where the values highlighted denote the best performance across sessions. The results revealed that when feedback was provided, the overall decoding performance improved by 4.45$\%$ and 0.28$\%$ for the VIS and VES groups, respectively. The performance improvement was marginal and not statistically significant (VIS: *p* = 0.2754, VES: *p* = 0.4316). Moreover, some participants did not benefit from the feedback considering their performance substantially decreased. The VES group also showed better performance than the VIS group in both the pre-feedback and feedback sessions, with differences of $12.05\%$ and $7.88\%$, respectively.

**Table 1:** Classification accuracy and Cohen’s kappa value of each participant, where bold values indicate best performance between sessions.

Group	Session	Participant										Accuracy	Kappa
		1	2	3	4	5	6	7	8	9	10		
VIS	Pre-feedback	68.26	75.95	**82.88**	**87.32**	53.74	51.85	87.27	70.79	**54.65**	**57.54**	69.23	0.3805
	Feedback	**92.91**	**94.04**	77.75	67.12	**68.91**	**59.23**	**93.55**	**83.89**	47.44	50.20	**73.68**	**0.4736**
VES	Pre-feedback	87.75	88.14	88.70	66.11	82.51	**77.75**	53.00	**88.12**	**82.18**	**98.74**	81.28	0.6256
	Feedback	**94.20**	**94.98**	**97.73**	**87.94**	**91.23**	69.40	**60.85**	81.71	40.41	97.42	**81.56**	**0.6312**

Providing feedback during the MI training led to a marginal improvement in decoding performance for both VIS and VES groups. Nevertheless, the VES group exhibited better performance than the VIS group.

## Discussion

Both groups exhibited lower levels of $\alpha$RP, particularly in the parietal lobe, which is responsible for integrating and processing sensory information from various modalities to produce appropriate motor responses. This indicates that the irrelevant cortical activity is suppressed during MI^3,18,39^. According to previous research^8^, the VES group was more attentive and experienced less difficulty during neurofeedback training compared to the VIS group. However, these assessments were subjective and not objectively analyzed. Neurofeedback training can modulate and enhance hemispheric asymmetry, resulting in improved motor task performance and promotion of motor learning^40,41^. In this study, left-sided frontal alpha asymmetry (FAA) was observed in both groups during the pre-feedback and feedback sessions and was associated with positive affect, approach motivation, and increased cognitive flexibility^42,43^. Interestingly, both groups showed a decreased in left-sided FAA during the feedback session. Nonetheless, the VES group still had higher left-sided FAA than the VIS group, thereby indicating that the VES group may be more effective at retaining positive emotions during a longer training process.

Although these findings may indicate successful neurofeedback training, the improvement in system classification accuracy was only marginal, which limits the practicality of the scheme, especially in motor rehabilitation applications. This can be attributed to the ceiling effect, in which the initial classification accuracy is already high, leaving little room for improvement^44^. In such situations, it may not be possible to achieve significant improvements in classification accuracy even with neurofeedback training. Another explanation could be insufficient training, considering that neurofeedback training requires time and practice^3,45^. It is possible that the training period was too brief or insufficient to achieve significant improvements in classification accuracy. Although there was a marginal improvement in the system classification accuracy after neurofeedback training, the VES group outperformed the VIS group in the feedback session, indicating the potential advantages of using multiple neurofeedback modalities. Incorporating electrotactile stimulation that is specific to the intended MI can enhance motor learning and improve the ability to generate mental representations of movement by reinforcing the associated neural pathways^37,46,47^.

The observed increase in $d_{GED}$ during the feedback session compared to the pre-feedback session may be attributed to personalized neurofeedback training, which allowed each participant to activate their subject-specific pathways and resulted in reduced inter-subject consistency of brain networks^14^. This can be attributed to the additional somatosensory information provided by electrotactile feedback, which reinforces the neural pathways associated with motor tasks^10,47^.

The combination of edge- and nodewise analyses can obtain a comprehensive understanding of changes in brain connectivity. Nodes with significant edges can have a greater influence on the overall network connectivity, suggesting that changes in their connectivity patterns may lead to significant changes in the network as a whole. The VIS group showed marginal variations in edge and node importance in the frontal and occipital lobes, suggesting that visual information remains influential in brain network organization. However, the potential benefits of visual feedback for cognitive and motor functions may be limited owing to the inability to improve the integration of information transfer between other brain regions. The VES group showed significant edges spanning from the occipital to the frontal lobe, and there was a noticeable increase in the node importance values at several electrode sites, including AF3, FCz, and C4, which indicate enhanced integration of information flow across these regions. This is critical for motor-related commands, motor planning, and higher-order cognitive functions. The decrease in importance values at O2 indicates that brain network reorganization favors neuroplasticity induced by electrotactile feedback over visual feedback considering haptic information is primarily processed in the motor cortex^46^. This finding is consistent with previous studies, which demonstrate that the addition of tactile feedback can reduce the cognitive workload associated with visually intensive tasks^10,11^. Interestingly, nodes C3, T7, and CP3 showed lower betweenness centrality but a higher degree, suggesting that they do not play a significant role in bridging the different regions of the network. Furthermore, the NBS analysis did not find significant differences between edges in this region, which may indicate that changes in connectivity patterns in these nodes do not contribute significantly to changes in the network as a whole.

Both the VIS and VES groups showed changes in their brain network MST structure after neurofeedback training, as demonstrated by longer diameter (*D*), lower leaf fraction (*Lf*), and lower maximum betweenness centrality (${\mathscr {C}}_{\textrm{B}_{max}}$). The observed changes indicate that the network became less centralized, adopting a more linear structure, indicating a decrease in the efficiency of information transfer across various brain regions^48^. Only the VES group showed significant changes in the ${\mathscr {C}}_{\textrm{B}_{max}}$ metric after training, which indicates that the transfer of information between brain regions became less reliant on the central nodes following visual-electrotactile feedback training. Note that the tree hierarchy (*Th*) values were close to 0.5, which suggests that the overall brain network organization remained star-like rather than line-like^30,33^. Brain networks are cost efficient and scale free, balancing strong local connectivity with efficient long-distance connections^24,49^. Although only ${\mathscr {C}}_{\textrm{B}_{max}}$ in the VES group showed a statistically significant change, the changes in the network structure observed in both groups may support the integration of sensory feedback and motor commands, and allow for less reliance on particular nodes. This suggests that these changes could facilitate multiple communication pathways.

In contrast to a similar study^37^, the global functional connectivity ($FC_{glob}$) did not exhibit significant changes across sessions. This study recorded brain signals immediately after the introduction of feedback, indicating that the long-term effects of feedback-induced neuroplasticity were not explored. It is also possible that neurofeedback is more effective at altering the organization of structural connections within the brain than the strength of functional connections during training^50,51^.

This study mainly included right-handed male participants within a limited age range, which may not be representative of the broader population. Research has shown that demographic differences such as age^52,53^, handedness^54^, and gender^55,56^ can affect the organization of the brain network, including its structural and functional connectivity and plasticity. Therefore, the findings of this study may not be generalizable to diverse populations. Additionally, the connectivity between various brain regions was estimated in this study using the weighted phase lag index (WPLI) method^57^. This approach was selected due to its beneficial characteristics, including its ability to handle non-linear interactions, robustness to signal artifacts, and insensitivity to volume conduction effects^26^. Nonetheless, similar to other phase-based connectivity estimation techniques, the WPLI has limitations. It does not offer information regarding the direction of interaction between two signals, and the precision of the connectivity estimation can be influenced by the frequency band selected for analysis. We acknowledge the potential for concurrent muscle activation and eye movements during the MI tasks, which could introduce motion artifacts and confounding factors in cortical activation patterns during MI. Since participant muscle activity and eye movement were not monitored in this study, difficulties arose in effectively eliminating these artifacts^58^. Consequently, these elements were not excluded from the analysis. Despite WPLI’s robustness against such artifacts, it remains important to acknowledge their potential presence to enhance the accuracy of functional connectivity estimation^59^.

While MST provides the benefit of reducing bias and improving comparability across studies, it does have drawbacks, such as its incapability to capture information about weaker connections in the network. This shortcoming can result in an oversimplified representation of the network structure and a loss of crucial information about less strongly connected nodes, particularly when the number of electrodes used is low^31^. Given that this study employed only 16 channels, there is a need to address this issue by expanding the number of channels. This adjustment would enable a more comprehensive investigation into the interactions among specific brain regions.

In summary, visual and visual-electrotactile stimulation feedback induced subject-specific changes in the activation patterns and network organization of the brain, while also promoting positive emotions through the maintenance of left-sided FAA. Visual-electrotactile stimulation feedback improved the information flow between the motor and cognitive regions and reduced cognitive workload. However, neither feedback modality significantly improved the classification accuracy. Nevertheless, multimodal neurofeedback holds promise for enhancing rehabilitation efficacy and should be considered in future interventions. Future studies should aim to recruit a more diverse sample population and employ complementary connectivity estimation techniques. Moreover, more advanced network construction methods should employed to enhance the representation of the brain network’s organization, by capturing information about weaker connections, which is particularly important when a limited number of electrodes is used, as this would also improve comparability across studies.

## Methods

**Figure 5:**
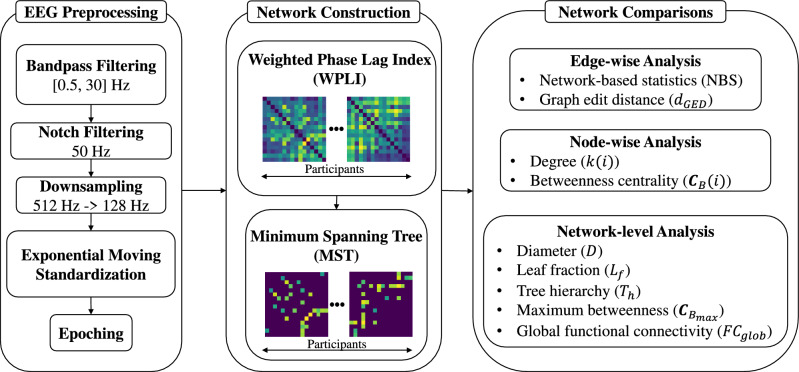
Overview of brain network analysis, which comprises EEG preprocessing, network construction, and network comparisons.

**Figure 6:**
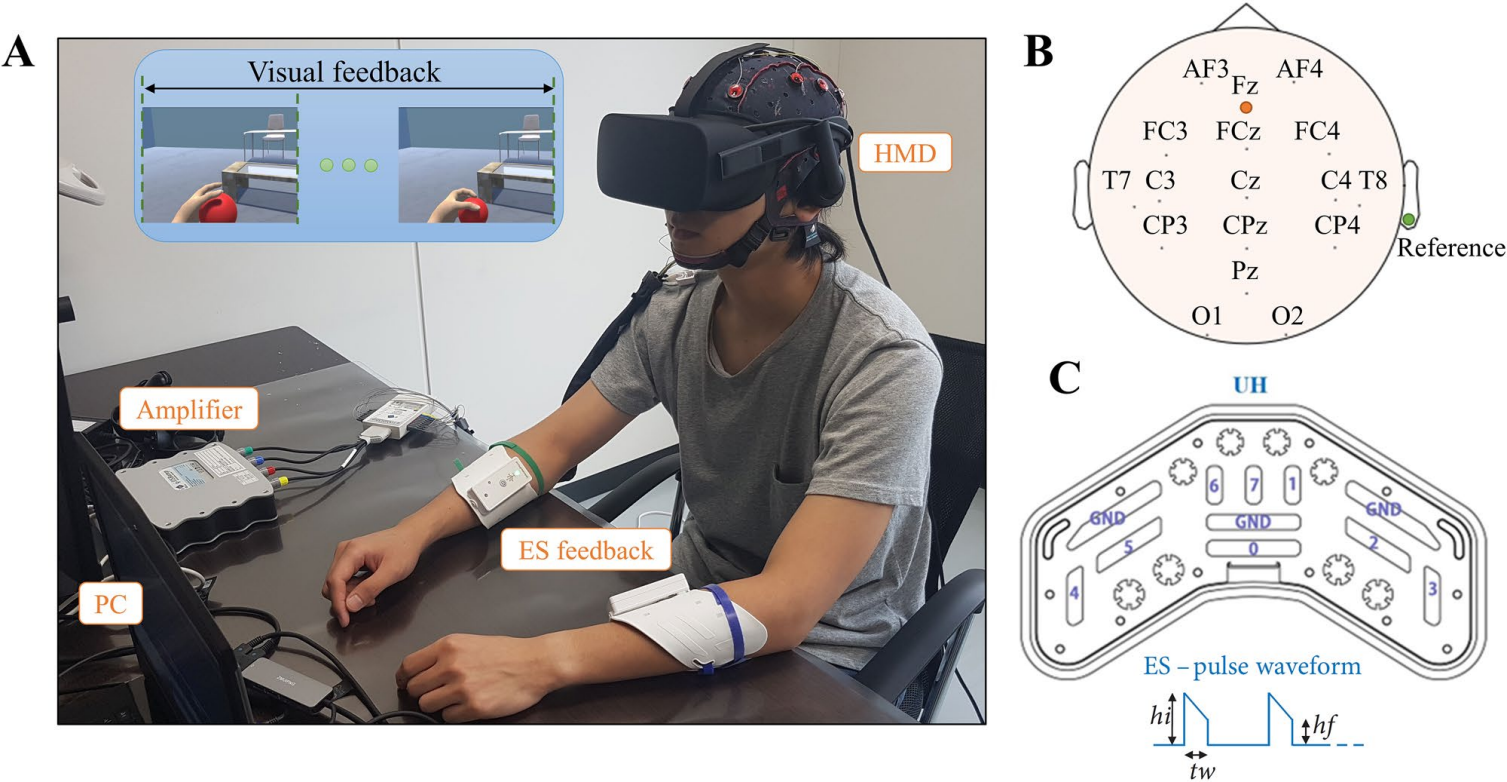
Experimental setup^8^. (**A**) Experimental equipment included an EEG amplifier, an HMD, two electrotactile stimulation (ES) devices, and a PC. (**B**) The electrode locations. (**C**) UnlimitedHand (UH) device used to provide electrotactile stimulation feedback.

**Figure 7:**
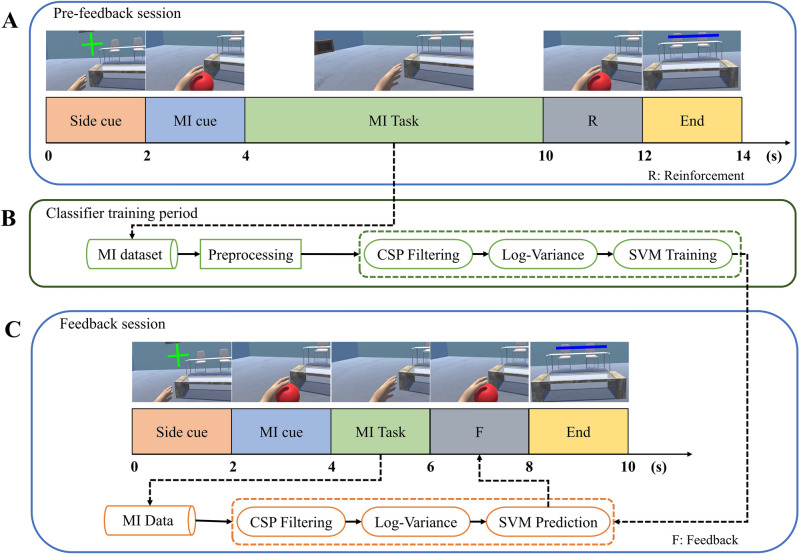
Timing scheme and flow chart of the experimental protocol^8^, where MI denotes motor imagery, CSP is the common spatial pattern algorithm, and SVM refers to support vector machine. (**A**) Pre-feedback session. (**B**) Classifier training period. (**C**) Feedback session.

Figure 5 shows the brain network analysis conducted in this study. First, EEG signals were collected using the experimental setup and timing scheme shown in Figs. 6 and 7, respectively, and preprocessed using filtering, downsampling, exponential moving standardization, and epoching. The resulting preprocessed signals were used to calculate the functional connectivity matrix within the $\alpha$ band using the WPLI. The MST algorithm was then applied to the matrix to construct a graphical representation of brain connectivity. Finally, various analyses, including node-, edge-, and network-wise analyses, were conducted on the graphs to identify any distinctive characteristics unique to each experimental condition.

Further details of each step are provided in the following sections.

### Data acquisition

#### Participants

Twenty naive participants in this study were equally divided into two groups: VIS (7 males, 3 females, 26.20 ± 3.37 years old, 1 left-handed), which only received visual stimulation, and VES (7 males, 3 females, 24.40 ± 5.16 years old, no left-handed), which received visual-electrotactile stimulation. None of the participants had any known neurological conditions, and all had normal or corrected-to-normal eyesight.

#### Experimental setup

Figure 6A shows the experimental setup. A 16-channel g.USBamp (g.tec Medical Engineering GMBH) amplifier with a 512 Hz sampling rate was used for brain activity recording. The locations of the wet active electrodes (AF3, AF4, FC3, FCz, FC4, C3, Cz, C4, T7, T8, CP3, CPz, CP4, Pz, O1, and O2) are shown in Fig. 6B, with the ground and reference electrodes placed at Fz and the right ear lobe, respectively. A head-mounted display (HMD), Oculus Rift (Facebook Technologies, LLC), with a display frequency of 90 Hz was mounted on the participants.

Each participant had two UH devices attached to the forearm for electrotactile stimulation (ES), as shown in Fig. 6C. ES intensity was calibrated before the experiment with a fixed pulse width (*tw*) of 0.2 ms and a voltage bootup (*hi*) level of 5V above the voltage level (*hf*). ES was performed using electrodes 0, 1, 3, 4, 6, and 7.

The participants were instructed to keep their head and eye movements to a minimum while the data were collected.

#### Experimental protocol

The experimental protocol was approved by the Ethics Committee of the School of Engineering, Tohoku University. Before the experiment, each participant provided informed consent, in accordance with the Declaration of Helsinki. They were notified that their identifying information and images may be presented in a publication. Privacy and confidentiality would be rigorously upheld, with all identifying information and images anonymized or pseudonymized to safeguard their identities.

The experiment consisted of two distinct sessions to train the classifier: a pre-feedback session, a feedback session, and a period between sessions. A complete explanation of the experimental protocol is provided in our previous work^8^.

Figure 7A shows the timing of the pre-feedback session, which involves randomly presenting a green cross (side cue) on either the left or right position at the start of each trial to indicate which side the MI task should be performed. When the virtual arm animation indicating the MI cue ended and the red ball vanished, the participant was instructed to begin the kinesthetic MI task repeatedly for 6 s. The participants in the VIS group were shown a virtual arm animation of the MI task during the reinforcement (R) period as a form of visual reinforcement. However, if the participants belonged to the VES group, both visual and electrotactile reinforcements were introduced during the R period. The blue line represents the end cue at the end of the trial. During the classifier training period, as shown in Fig. 7B, common spatial pattern (CSP)^60^ filtering and log-variance computations were used to extract features from the samples collected during the pre-feedback session. These features were used to train a support vector machine (SVM) whose confidence score is used to determine the feedback level for the feedback session. Figure 7C shows the timing scheme for the feedback session. The only differences between the feedback session and the pre-feedback session was the decrease in the MI period from 6 s to 2 s and the replacement of the feedback (F) period with the R period. Similarly, the MI virtual arm animation was shown as visual feedback to the participants in the VIS group. However, if the participant belonged to the VES group, visual-electrotactile feedback was provided.

The experiment comprised two runs for each MI task in each session, with 40 trials (20 on each side) in each run. The participants were given a 5 min break between sessions and a 1 min break between runs.

### Preprocessing

The EEG signals were filtered using an eighth-order Butterworth bandpass filter with cutoff frequencies of [0.5, 30] Hz, and a fourth-order Butterworth 50 Hz notch filter. The signals were later downsampled by a factor of 4 from 512 to 128 Hz and scaled using exponential moving standardization. No artifacts were removed.

Only the first 2 s of the MI period, which lasted for 6 s in the pre-feedback session, were retrieved for analysis, as the MI period of the feedback session was 2 s. This process was performed to balance the samples.

### Power spectral

To examine how different types of feedback influence the frequency content of brain signals, the power spectral density (PSD) was calculated using the multitaper method with a frequency resolution of 0.5 Hz. The calculation of frontal alpha symmetry (FAA), a helpful marker for emotional and cognitive processing, involves the use of the logarithmic ratio of $\alpha$-band PSD in the right and left frontal lobes, expressed as:

1 $$\begin{aligned} FAA=\ln \left( \frac{\textrm{PSD}^{\alpha }_{right}}{\textrm{PSD}^{\alpha }_{left}}\right) , \end{aligned}$$

where $\ln (\cdot )$ is the natural logarithmic function and $\textrm{PSD}^{\alpha }$ is the PSD of $\alpha$ band. A positive FAA score suggests greater $\alpha$ activity in the right frontal region relative to the left, which is associated with withdrawal-related emotions such as anxiety and sadness. In contrast, a negative FAA score suggests greater $\alpha$ activity in the left frontal region relative to the right, which is associated with approach-related emotions, such as happiness and enthusiasm^42,43,61^. Relative power refers to the alteration in band power during an event compared to the baseline period^17^, expressed as:

2 $$\begin{aligned} \textrm{RP}=\frac{\textrm{PSD}_{x}-\textrm{PSD}_{baseline}}{\textrm{PSD}_{baseline}}. \end{aligned}$$

### Network construction

#### Weighted phase lag index

Neural oscillations of two or more brain areas can synchronize or fire at the same time and frequency when performing a task or analyzing the same information. One approach for measuring the level of synchronization between various brain areas is the phase-lag index (PLI)^57^. The index is defined as:

3 $$\begin{aligned} PLI=|E\{sgn({\mathfrak {J}}\{\textrm{X}\})\}|, \end{aligned}$$

where ${\mathfrak {J}}\{\textrm{X}\}$ is the imaginary component of the cross-spectrum, *sgn* is the signum function, $E\{\cdot \}$ is the expected value symbol, and $|\cdot |$ is the absolute value symbol. Because PLI ignores the phase synchronization at 0 and $\pi$, it is relatively insensitive to common sources of artifacts and the volume conduction effect. However, this does not account for the strength of the phase differences between the two signals. Small perturbations can cause a phase lag to lead, and vice versa, resulting in spurious measures of phase synchronization. In contrast, the weighted PLI (WPLI) considers the magnitude of the imaginary component of the cross-spectrum to weight the impact of the observed phase leads and lags^59^. Thus, the WPLI can detect weak synchronization more effectively and is less susceptible to sources of uncorrelated noise than the PLI. WPLI is defined as:

4 $$\begin{aligned} WPLI=\frac{|E\{{\mathfrak {J}}\{\textrm{X}\}\}|}{E\{|{\mathfrak {J}}\{\textrm{X}\}|\}}=\frac{|E\{|{\mathfrak {J}}\{\textrm{X}\}| sgn({\mathfrak {J}}\{\textrm{X}\})\}|}{E\{|{\mathfrak {J}}\{\textrm{X}\}|\}}. \end{aligned}$$

The WPLI ranged from 0 to 1, where 0 indicates no phase synchronization and 1 indicates perfect phase synchronization. The index was calculated using the MNE-Connectivity Python package^62^. Because the phase synchronization measures, including the WPLI, were frequency-dependent, they were often calculated separately for different frequency bands. The WPLI value was determined for each EEG electrode pair, resulting in a adjacency matrix (*A*) of size 16 $\times$ 16 for each frequency band of interest.

Although the WPLI is a widely used phase-based connectivity estimator, it has limitations that are common to other methods in this category. First, it is nondirectional (i.e., it does not indicate the direction of the interaction between two signals). Second, the choice of the frequency band can affect the accuracy of the connectivity estimate, making it sensitive to the analysis frequency range. These issues should be considered when interpreting the results obtained with the WPLI, considering they may affect the reliability and validity of the obtained functional connectivity estimates^26^.

#### Minimum spanning tree

Graph-theoretical methods, which can investigate network topology, are frequently employed to study the brain^21^. However, research on brain networks has produced inconsistent findings, and it is difficult to reach an agreement on how the brain’s structural and functional networks are organized. Recently, it was proposed that MST may enhance study comparability^29,30^.

A spanning tree is a subset of edges that connects all nodes in a graph without forming any cycles. The MST is defined simply as a spanning tree with the minimum possible total edge weight, which can be obtained using Prim’s algorithm^63^ or Kruskal’s algorithm^64^. The connection strength between each pair of electrodes in the connectivity matrix is represented by the edge weight, which can be considered as the inverse distance. Therefore, to obtain MST for brain network analysis, the weights are either multiplied by or raised to the power of − 1; this is also known as the maximum spanning tree.

Although MST reduces bias and improves comparability across studies, it only captures information about the strongest connections in the brain network and ignores weaker connections. This approach can oversimplify the network structure and lead to the loss of significant information regarding less strongly connected nodes^31^.

### Network comparisons

#### Edge-wise analysis

The analysis of connections between brain regions, known as edgewise analysis, can provide valuable insights into patterns of communication and integration between brain areas, including identifying critical connections for specific brain processes and behaviors.

Network-based statistics (NBS) are statistical tools that can be used to identify patterns or sets of edges that differentiate brain functional connectivity between two conditions^27^. NBS reduces the likelihood of false discoveries using a permutation-based approach, thereby reducing the family wise error rate associated with multiple statistical tests.

The graph edit distance ($d_{GED}$) was used to quantify the dissimilarity between the two graphs. In particular, $d_{GED}$ is calculated as the minimum number of operations required to transform one graph ($g_1$) into another that is isomorphic to the second graph ($g_2$). $d_{GED}$ is calculated as:

5 $$\begin{aligned} d_{GED}\left( g_1, g_2\right) =\min _{\left( e_1, \ldots , e_k\right) \in {\mathscr {P}}\left( g_1, g_2\right) } \sum _{i=1}^k c\left( e_i\right) , \end{aligned}$$

where ${\mathscr {P}}\left( g_1, g_2\right)$ is the collection of edit paths, and $c\left( e_i\right)$ is the cost of the edit operation *e*. *e* includes the insertion, removal, or deletion of nodes or edges. A high $d_{GED}$ indicates that the two graphs are highly dissimilar.

#### Node-wise analysis

In the nodewise analysis, graph metrics are calculated for each node in a graph, and the resulting metric values for each node are compared between the graphs. This type of analysis offers several advantages, such as the ability to examine a wider range of graph features, access more data to compare different conditions, and identify specific brain regions where differences between conditions occur, rather than simply determining whether differences exist^25^.

The degree is a measure of the number of edges connected to a specific node. Nodes with high degrees are referred to as hubs and are considered to process and integrate information within the brain network. The degree of a node is given as:

6 $$\begin{aligned} k(i)=\sum _{j=1, j \ne i}^N A_{i j}, \end{aligned}$$

where *A* is an adjacency matrix and *N* is the number of nodes.

The betweenness centrality is used to quantify the extent to which a given node serves as a bridge or intermediary between other nodes. It counts the number of shortest routes connecting a particular node to other nodes in pairs. In brain network analysis, high-betweenness centrality nodes accommodate information integration and transfer throughout the network.

7 $$\begin{aligned} {\mathscr {C}}_{\textrm{B}}(i)=\frac{1}{(N-1)(N-2)} \sum _N^{h=1, h \ne j} \sum _N^{j=1, j \ne i} \frac{\sigma _{h j}(i)}{\sigma _{h j}}, \end{aligned}$$

where $\sigma _{h j}$ corresponds to the number of shortest paths that exist between nodes *h* and *j*, and $\sigma _{h j}(i)$ represents $\sigma _{h j}$ that passes through node *i*.

#### Network-level analysis

Network-level analysis is a method for determining how the brain network is topologically organized in the global scale.

Diameter refers to the greatest distance between any two nodes normalized by the number of edges. A low diameter signifies more effective information flow across brain areas, whereas a high diameter implies a loss in overall efficiency.

8 $$\begin{aligned} D=\frac{d}{M}, \end{aligned}$$

where *d* is the greatest distance between any two nodes and *M* is the number of edges.

Leaf fraction is a metric for measuring centrality based on the number of nodes with only one connection, called leaves. A lower leaf fraction suggests that the network exhibits a linear topology where communication is less centralized. In contrast, a high leaf fraction network features a star-like topology, where communication is highly reliant on hub nodes.

9 $$\begin{aligned} Lf=\frac{L}{M}, \end{aligned}$$

where *L* is the number of leaves.

The tree hierarchy quantifies the trade-off between large-scale integration in the MST and the overload of the central nodes^33^. The value tends towards 0 and 0.5 for the line and star topologies, respectively.

10 $$\begin{aligned} Th=\frac{L}{2M{\mathscr {C}}_{\textrm{B}_{max}}}, \end{aligned}$$

where ${\mathscr {C}}_{\textrm{B}_{max}}$ is the maximum betweenness centrality calculated by finding the maximum value from Eq. (7).

In this study, global functional connectivity is defined as the sum of the edge weights of the MST of an adjacency matrix (*A*). Strong functional connectivity in the brain is often linked to effective integration and communication across various brain regions^65^. This indicates that the brain functions in a coordinated and cohesive manner.

11 $$\begin{aligned} FC_{glob}=\sum _{e \in E(T)} w(e), \end{aligned}$$

where *T* is the MST of *A*, *E*(*T*) is the set of edges in *T*, and *w*(*e*) is the weight of the edge *e*.

The visualizations of brain connectivity were produced utilizing the Python libraries Nilearn^66^ and Networkx^67^.

### Decoding

Previous studies^8,68,69^ demonstrated the superior performance of deep learning models over traditional methods such as CSP in decoding MI from the Tohoku University MI dataset. Therefore, this study employed a deep learning model. However, these studies did not evaluate the classification accuracy within the same session or focused only on decoding data from the VES group. Thus, the present study compared the intrasession performance of the VIS and VES groups to investigate how different feedback modalities affected the MI performance.

This study employed ShallowConvNet^38^ for the binary classification of MI (left and right) tasks. The model was inspired by the filter bank CSP (FBCSP)^70^, which effectively extracts spatiotemporal features by dividing the EEG signal input into multiple sub-bands. The first two layers of ShallowConvNet perform spatial and temporal filtering, which is analogous to the bandpass and spatial filtering processes of FBCSP. Similarly, logarithmic activation, average pooling, and batch normalization mirror the log-variance calculation of FBCSP. In contrast to FBCSP, ShallowConvNet enables joint optimization of all feature extractions and classifications by integrating them into a single end-to-end model.

The classification model was developed using the Keras API in the TensorFlow library, and trained on an Intel Core i7 CPU and an NVIDIA GeForce GTX 1070 GPU. The model was fitted using the Adam optimizer with a learning rate of $1e-3$, batch size of 32, and dropout probability of 0.5. Using 10 iterations of stratified tenfold cross-validation with different randomizations for each iteration, the samples were split into training and test sets without data leakage, thereby allowing the experiments to be evaluated 100 times on different sets of training and test samples to reduce bias. In addition, 1 s time windowing with 90% overlap (i.e., a stride of 0.1 s) was used to augment the samples. When the training loss did not improve for 10 epochs, the training was terminated (i.e., early termination of 10 epochs).

### Statistical analysis

The Wilcoxon signed-rank test was used to determine significant differences. The significance level was adjusted for multiple comparisons using a false discovery rate (FDR) correction^71^ with an adjusted significance level of 0.05.

## Data Availability

The datasets generated during and/or analyzed during the current study are available from the corresponding author on reasonable request.
